# Multimodal rehabilitation in PLP1-associated spastic paraparesis: a case report with clinical and biomechanical outcomes

**DOI:** 10.3389/fresc.2026.1837911

**Published:** 2026-06-16

**Authors:** Mirjam Bonanno, Mauro Botindari, Antonino Lombardo Facciale, Silvia Migale, Angelo Quartarone, Rocco Salvatore Calabrò

**Affiliations:** IRCCS Centro Neurolesi Bonino-Pulejo, Messina, Italy

**Keywords:** Alter-G, case report, EXOPULSE Mollii Suit, gait analysis, neurorehabilitation, Pelizaeus–Merzbacher disease, PLP1, spastic paraparesis

## Abstract

**Background:**

PLP1-related disorders, including the Pelizaeus–Merzbacher disease spectrum and X-linked spastic paraplegia type 2, are rare hypomyelinating conditions characterized by progressive gait impairment, lower limb spasticity, and motor disability. Evidence supporting rehabilitation strategies in these disorders remains limited, particularly for technology-assisted interventions.

**Case presentation:**

We report the case of a 29-year-old man with progressive PLP1-related spastic paraparesis, prominent gait impairment, recurrent falls, lower limb spasticity, and preserved independence in activities of daily living. Genetic testing identified a hemizygous PLP1 variant, classified as likely pathogenic. The patient underwent a 20-session outpatient rehabilitation program organized into two sequential blocks: 10 sessions of anti-gravity treadmill training (Alter-G), followed by 10 sessions combining Alter-G and EXOPULSE Mollii Suit stimulation.

**Methods and outcomes:**

Clinical, gait analysis, and posturographic outcomes were collected at baseline (T0), after the first block (T1), and after completion of the full intervention (T2). Across the intervention period, balance, lower limb strength, and spasticity showed overall improvement, whereas timed mobility and endurance displayed an initial worsening at T1 followed by partial recovery at T2. Instrumental gait analysis revealed marked step-length reduction after the first block, with substantial recovery by T2, alongside side-specific changes in gait quality indices. Static posturography showed progressive normalization of foot load distribution under eyes-open conditions, while residual asymmetry persisted with eyes closed. Fatigue scores, assessed during the second block only, improved over time.

**Conclusion:**

This case suggests that sequential technology-assisted rehabilitation combining body weight-supported treadmill training and wearable electrical stimulation is feasible in PLP1-related spastic paraparesis and may be associated with improvements in selected domains, including balance, strength, spasticity, and selected gait parameters. Given the single-case design, multimodal treatment structure, and potential confounding factors, these findings should be considered exploratory and hypothesis-generating.

## 1 Introduction

Pelizaeus–Merzbacher disease (PMD) and X-linked spastic paraplegia type 2 (SPG2) belong to a phenotypic spectrum of PLP1-related disorders caused by pathogenic variants in the *PLP1* gene. This gene encodes proteolipid protein 1, a key structural component of central nervous system myelin (1). Clinical severity is heterogeneous, ranging from severe early onset hypomyelinating phenotypes to milder forms characterized predominantly by progressive spastic paraparesis (1, 2). In milder adult phenotypes, gait impairment, lower limb hypertonia, postural instability, and fatigability may represent the most functionally relevant manifestations. Despite the progressive and disabling nature of these motor symptoms, the rehabilitation literature on PLP1-related disorders remains extremely limited (3, 4). Most published reports have focused on diagnostic aspects, genotype–phenotype correlation, anesthesiologic management, or pediatric supportive care (1, 3, 5, 6). In particular, evidence on structured rehabilitation programs in adults with PLP1-related spastic paraparesis is scarce, and systematic descriptions of protocol intensity, progression, and multidomain outcomes are largely lacking. This gap is clinically relevant because patients with slowly progressive phenotypes may retain functional reserve and may therefore benefit from targeted, intensive, and technology-assisted rehabilitation strategies.

Among available approaches, body weight-supported gait training may allow intensive locomotor practice while reducing the biomechanical cost of walking and the risk of falls (7). In parallel, wearable electrical stimulation systems such as the EXOPULSE Mollii Suit have been proposed as adjunctive tools to modulate spasticity and facilitate functional movement, although evidence remains heterogeneous and condition specific (8, 9). To date, the combined use of anti-gravity treadmill training and wearable electrical stimulation has not been described in adults with PLP1-related spastic paraparesis.

Here, we report the clinical and instrumental trajectory of a patient with PLP1-related spastic paraparesis who completed a 20-session technology-assisted rehabilitation program consisting of anti-gravity treadmill training followed by combined anti-gravity treadmill and wearable electrical stimulation. The primary aim of this case report is to document feasibility and to describe multidomain outcome trajectories across three timepoints (T0, T1, T2). We additionally discuss the potential clinical relevance of this approach in the context of this rare condition, while recognizing the exploratory nature of single-case observations.

## 2 Case presentation

### 2.1 Patient history

A 29-year-old man, born in Ukraine and currently living in Italy, was referred to our Neurorehabilitation Unit in October 2025 for evaluation of progressive spastic paraparesis with marked gait impairment and recurrent falls. According to the available clinical history, he had shown toe-walking and frequent falls since early childhood, with progressive worsening over time. He had previously undergone several neurological evaluations in Ukraine and Italy. He underwent functional assessment and rehabilitation planning at our unit. At the time of admission, the etiological work-up was still being completed. A molecular diagnosis of a PLP1-related disorder was subsequently confirmed during follow-up (genetic report finalized on 20 November 2025) and was considered consistent with the clinical phenotype (see Table 1).

**Table 1:** Timeline of clinical history, diagnostic milestones, and intervention phases.

Period	Event
Early childhood	Onset of toe-walking and recurrent falls (per clinical history).
2018	Brain MRI: subtle white matter signal abnormalities; evoked potentials supported central pathway involvement.
2021	Functional surgery: neurotomy of obturator motor branches targeting hip adductors.
2024	IncobotulinumtoxinA injections to gastrocnemius medialis/lateralis and soleus bilaterally (total 300 U).
2025 (October)	Referral to Neurorehabilitation Unit; baseline multidomain assessment (T0) and rehabilitation planning.
2025 (November)	Genetic report finalized: hemizygous PLP1 variant classified as likely pathogenic.

No additional relevant family history was available from the clinical records. His parents were reported as non-consanguineous, and one sister was reportedly unaffected.

#### 2.2 Diagnostic assessment

Before referral to our center, the patient had already undergone an extensive neurological and instrumental diagnostic evaluation. Electromyography was reportedly within normal limits, whereas somatosensory evoked potentials were described as abnormal. Brain magnetic resonance imaging performed in 2018 showed subtle white matter signal abnormalities, reported as blurred hyperintensity in the bilateral paratrigonal regions and centrum semiovale. Evoked potential studies supported central pathway involvement. Lower limb somatosensory evoked potentials showed normal peripheral responses, absence of the right cortical response, and a low-amplitude left cortical response with prolonged latency, consistent with dysfunction of the central somatosensory pathways involving the lower limbs. Visual evoked potentials were abnormal, predominantly on the left side, indicating visual pathway dysfunction of mainly axonal type. Brainstem auditory evoked potentials were within normal limits.

As part of the pre-referral diagnostic work-up, a previous clinical–instrumental gait assessment suggested that spasticity coexisted with compensatory mechanisms supporting progression and balance. In particular, the dorsiflexion deficit was considered mainly related to triceps surae shortening, more pronounced on the right side, with only a minor contribution from intermittent pathological activation during swing. Excessive hip adduction, worsening with increasing speed and step length, was considered compatible with adductor overactivity.

Genetic testing for hereditary spastic paraplegia had also been performed before admission to our center on genomic DNA extracted from peripheral blood. Targeted resequencing was conducted on an Illumina platform using probes based on Twist Human Core Exome technology. Variant annotation was performed with eVAI v3.7, and interpretation followed ACMG criteria. The analysis identified a hemizygous *PLP1* variant (NM_000533.5: c.365A > G; p.Lys122Arg), classified as likely pathogenic, with CADD 23.6 and absent frequency in gnomAD v4.0.1. The molecular finding was considered likely to explain the clinical phenotype. The genetic report was validated on 20 November 2025.

### 2.3 Previous therapeutic management

Before referral to our center, the patient had undergone regular physiotherapy and botulinum toxin treatment as part of long-term spasticity management. In 2021, he underwent functional surgery described as neurotomy of the motor branches of the obturator nerve targeting the hip adductors.

On 20 February 2024, he received incobotulinumtoxinA injections (Xeomin), consisting of 50 U each into the gastrocnemius medialis, gastrocnemius lateralis, and soleus muscles bilaterally, for a total dose of 300 U. In the following weeks, he reported increased fatigability during rehabilitation, without substantial qualitative gait improvement. This was cautiously interpreted as a potential reduction in compensatory propulsion mechanisms. In chronic spastic gait, plantarflexor activity may support push-off/vaulting to aid swing clearance; therefore, reducing plantarflexor overactivity/strength could transiently increase perceived effort and fatigue without clear qualitative gait improvement. This interpretation is speculative due to the observational design and lack of objective post-injection data.Despite these previous interventions, gait impairment and instability persisted. Given the limited benefit obtained with conventional rehabilitation approaches, a technology-assisted rehabilitation program was considered.

### 2.4 Clinical presentation at admission

At admission to our center, the patient reported persistent lateral trunk bending during walking, difficulty in bilateral hip and knee flexion during swing, and multiple daily falls.

Despite these limitations, he maintained a relatively high functional status. He walked independently indoors and outdoors, remained independent in activities of daily living and instrumental activities of daily living, drove a car, and used public transportation without assistance. He climbed stairs using a handrail. Standardized functional scores were: Rivermead Mobility Index, 13/15; Functional Ambulation Category, 5/5; and Walking Handicap Scale, 5/6. He occasionally used a forearm crutch for safety, although he preferred not to rely on it. At the neurological examination, gait was spastic paraparetic (scissor-like) with compensatory strategies. Cranial nerve examination showed mild left convergent strabismus and nystagmus on left lateral gaze, whereas the remaining cranial nerves were unremarkable. Distal hypotrophy was reported, particularly affecting intrinsic hand muscles and ankle dorsiflexors. Bilateral pes cavus was present. Lower limb weakness predominantly involved the quadriceps and tibialis anterior muscles (right > left). Muscle tone was increased in both lower limbs, particularly at the ankles (right > left). Deep tendon reflexes were brisk in all limbs, with bilateral Babinski sign, bilateral Hoffmann sign, and prominent ankle clonus on the right. Sensory examination (light touch and vibration) was normal. Mild bilateral dysmetria on finger-to-nose testing had been reported in a previous neurological examination.

### 2.5 Functional diagnosis and rehabilitation rationale

A focused gait and functional assessment performed at our center described reduced hip and knee flexion bilaterally, lumbar hyperlordosis, and pelvic/trunk displacement more evident on the right side. Rectus femoris hypertonia was noted bilaterally, without significant hamstring shortening. Hip flexor tightness was associated with compensatory pelvic tilt. A reducible bilateral equinus pattern was observed, partially compensated by foot pronation, with clonus elicitable at medium threshold.

Overall, the functional diagnosis was consistent with spastic paraparesis characterized by bilateral hip flexion deficit, bilateral equinus, impaired gait efficiency, and persistent compensatory strategies affecting balance and progression. Based on the chronicity of gait impairment, the persistence of falls, and the limited response to previous conventional rehabilitation strategies, a technology-assisted rehabilitation program was proposed, combining task-specific locomotor training with progressive body-weight support modulation and, in a second phase, wearable electrical stimulation.

## 3 Materials and methods

### 3.1 Study design

This work is a single-patient case report describing clinical and instrumental changes across three assessment timepoints: baseline (T0), after the first treatment block (T1), and after completion of the full intervention program (T2). Outcome data were extracted from routine clinical and instrumental assessments performed as part of the patient's rehabilitation pathway. The manuscript was prepared in accordance with the CARE 2013 reporting guidelines for case reports (10) (See Supplementary Material 1).

The rehabilitation program was delivered in an outpatient clinical rehabilitation setting. Written informed consent was obtained for anonymized use of clinical data for research and publication purposes, in accordance with institutional policies and the Declaration of Helsinki (IRCCS-ME-45/2020).

### 3.2 Intervention

The rehabilitation program consisted of 20 sessions divided into two consecutive 10-session blocks.

**Figure 1:**
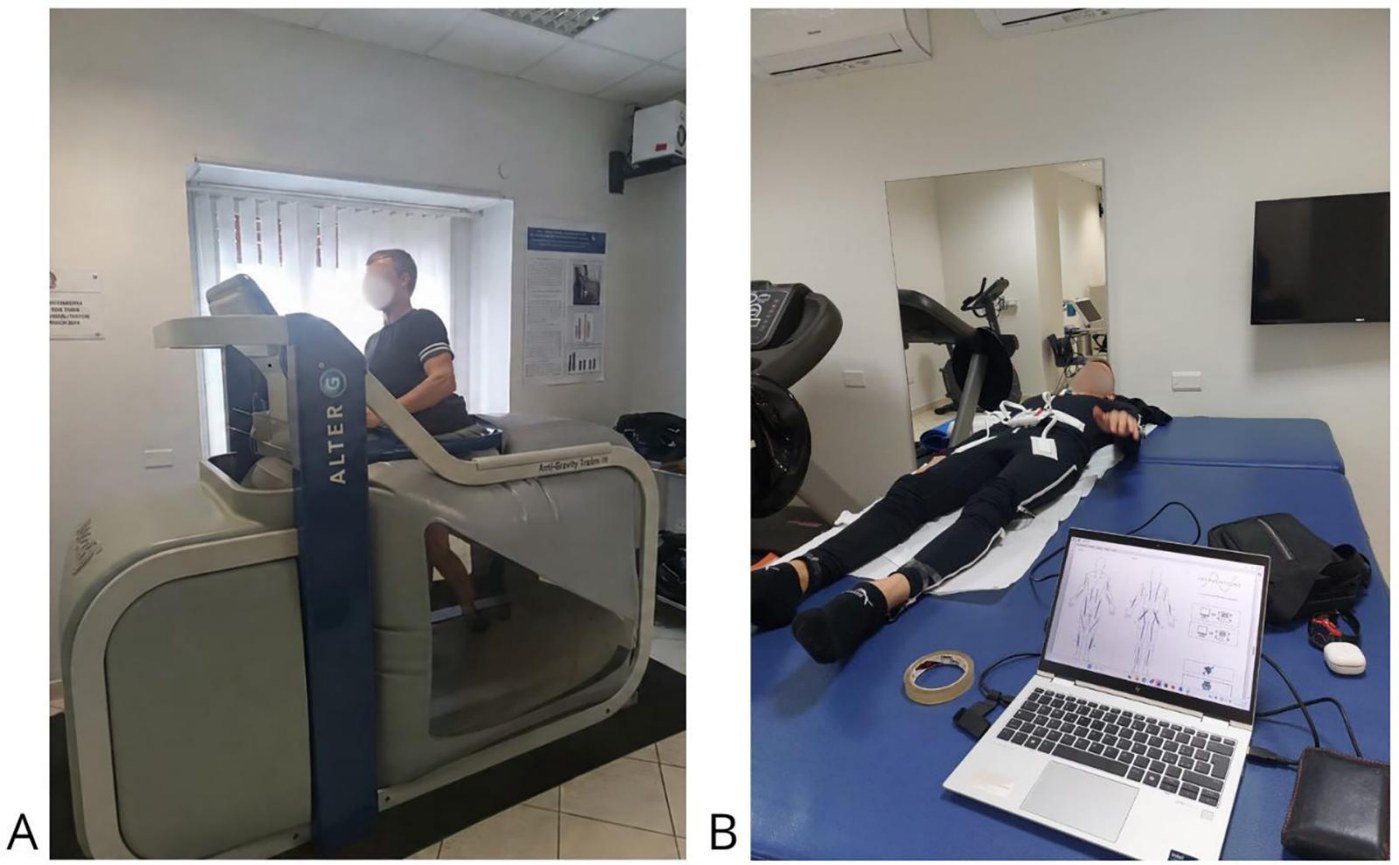
The patient during Alter-G training **(A)** and while using the EXOPULSE Mollii Suit **(B)**.

Block 1 (sessions 1–10): Alter-G-only training. During the first block, the patient underwent gait training using an anti-gravity treadmill (Alter-G Anti-Gravity Treadmill, model M320, Alter-G Inc., Fremont, CA, USA). This lower-body positive pressure system allows controlled unloading of body weight while preserving a relatively physiological gait pattern and reducing fall risk and joint loading (see Figure 1, panel A).

Block 2 (sessions 11–20): combined Alter-G plus EXOPULSE Mollii Suit. During the second block, Alter-G training was maintained and combined with the EXOPULSE Mollii Suit (Exoneural Network AB, Danderyd, Sweden), a full-body garment with integrated electrodes designed to deliver low-frequency electrical stimulation to selected muscle groups (see Figure 1, panel B).

During the second intervention block, the EXOPULSE Mollii Suit was delivered according to the stimulation parameters reported in the Supplementary Material. Stimulation intensity was programmed using the device ordinal scale expressed as pulse width, where 1 corresponds to 25 µs and each incremental unit increases pulse width by 5 µs (maximum 30 = 170 µs). In the adopted program, stimulation was distributed bilaterally across upper limb, trunk, and lower limb regions (anterior and posterior views), including shoulder/upper chest, upper arm, forearm, abdominal wall and lateral trunk, gluteal and hip regions, anterior and posterior thighs, and calf regions. The highest stimulation targeted the tibialis anterior (TA), with right TA = 21 (125 µs) and left TA = 18 (110 µs). Full electrode-by-electrode settings (region, side, ordinal value, and corresponding pulse width) are provided in Supplementary Table S1).

Alter-G training was performed at least three times per week. Each session lasted approximately 60 min, including device setup, patient preparation, active training, and final removal from the system. The effective walking time on the treadmill was approximately 30–40 min per session, delivered either as continuous walking. During the combined block, EXOPULSE Mollii Suit sessions were added on alternate days according to clinical planning. A physiotherapist continuously supervised each session. No physical assistance was required for stepping, and the patient used the treadmill's handrails as needed for balance (particularly during speed/incline increments). Across the intervention period, Alter-G parameters were progressively modified to increase task demand, with body-weight support gradually reduced from 80% to 55%, treadmill incline increased from 0° to 2°, and speed increased from 0.5 to 0.7 km/h, according to patient tolerance and safety.

In addition to the preplanned clinical and instrumental assessments, several session-level parameters were monitored during Alter-G training in order to characterize physiological response and tolerability. These included peripheral oxygen saturation (SpO₂), heart rate, systolic and diastolic blood pressure, perceived exertion rating (RPE), distance covered per session, and estimated energy expenditure. These variables were summarized descriptively across the two treatment blocks and used to provide additional information on the feasibility and physiological burden of the intervention.

In particular, the RPE was used to guide the progression of the task demand on the Alter-G. When the patient reported a low RPE (approximately 1–2), the training load was progressed by reducing body-weight support (i.e., decreasing the percentage of unweighting) and/or increasing treadmill speed and incline. Conversely, when RPE was moderate-to-high or when gait stability decreased, parameters were maintained or temporarily adjusted to ensure tolerability and safety.

### 3.3 Outcome measures

The patient underwent a multidomain assessment at T0, T1, and T2, including clinical, instrumental gait, and posturographic measures.

Clinical measures included (see Supplementary Table S2 for the description of the used tools):

- Falls Efficacy Scale–International (FES-I), to assess fear of falling;
- Berg Balance Scale (BBS), to assess static and dynamic balance;
- Timed Up and Go (TUG), to assess functional mobility;
- Motricity Index (MI), to assess lower limb strength;
- Ashworth Scale (AS), to assess spasticity;
- 10 Meter Walk Test (10 MWT), to assess short-distance walking performance;
- 6 Min Walk Test (6 MWT), to assess walking endurance.

Fatigue measures included the Visual Analog Fatigue Scale (VAFS) and Fatigue Severity Scale (FSS), but these were assessed only during the second treatment block and therefore were not available at baseline.

Instrumental gait analysis was performed using the BTS Gaitlab marker-based motion analysis system. Data were acquired and processed in BTS SMART-Clinic using the DAVIS Heel multifactorial gait analysis protocol (Newington marker set) (11), with standard anthropometric scaling. Spatiotemporal and gait quality indices (e.g., step length/width, cadence, gait profile score, gait deviation index) were extracted at T0, T1, and T2 and interpreted against normative data from a healthy adult cohort (40 participants; 28 males; 12 females; 18–40 years) (12, 13). Postural control was assessed through static posturography using a Zebris platform (Zebris Medical GmbH, Germany), including foot load distribution, confidence ellipse, center-of-pressure length, and average center-of-pressure velocity under eyes-open and eyes-closed conditions.

### 3.4 Data analysis

Given the descriptive single-case design, no inferential statistics were performed. Outcomes were analyzed descriptively across timepoints, focusing on direction of change, side-specific trends, and the relative evolution of clinical and instrumental measures during the two intervention phases.

For selected clinical outcomes, changes were additionally contextualized against published Minimal Detectable Change at the 95% confidence level (MDC95) (or MDC95 derived from the Standard Error of Measurement–SEM-derived MDC95) values as an estimate of measurement error; these comparisons were exploratory and are detailed with references in the Supplementary Table S3. Because disease-specific measurement properties for PLP1-related spastic paraparesis are lacking and most published MDC/SEM estimates derive from stroke or other conditions (14–19), measurement-error contextualization was considered exploratory.

## 4 Results

### 4.1 Clinical outcomes

Clinical outcomes showed a heterogeneous but overall favorable pattern across timepoints (see Table 2 and Supplementary Figure S1). Balance improved progressively, with the Berg Balance Scale increasing from 35 at baseline to 39 at T2. Lower limb strength also improved, particularly on the right side, where the Motricity Index (MI) increased from 44 to 56, while the left side improved from 52 to 56. Spasticity decreased bilaterally only at the end of the program, with Ashworth Scale (AS) scores decreasing from 6 to 5 on both sides. By contrast, gait-related performance measures showed a non-linear trajectory: 10 m walking test (10MWT) and Timed-Up-Go (TUG) worsened at T1 and then partially recovered at T2. Walking endurance showed a similar course, with the 6 min walking test (6MWT) decreasing from 150 m to 125 m at T1 and then improving to 145 m at T2. Fear of falling remained unchanged across all assessments (Falls Efficacy Scale—International, FES-I = 16). Fatigue measures, collected only during the second treatment block, improved markedly, with Fatigue Severity Scale (FSS) decreasing from 24 to 9 and Visual Analog Fatigue Scale (VAFS) from 3 to 0.

**Table 2:** Clinical outcomes variations across baseline (T0), post-Alter-G only training (T1) and post-combined training (T2).

Outcome	Side	Direction of improvement	T0	T1	T2
FES-I	–	Lower is better	16	16	16
Berg Balance Scale (BBS)	–	Higher is better	35	36	39
10-Meter Walk Test (s)	–	Lower is better	11.22	17.52	12.72
6-Minute Walk Test (m)	–	Higher is better	150	125	145
Fatigue Severity Scale (FSS)	–	Lower is better	–	24	9
Visual Analog Fatigue Scale (VAFS)	–	Lower is better	–	3	0
Timed Up and Go (TUG, s)	Right	Lower is better	19.87	23.45	19.83
Timed Up and Go (TUG, s)	Left	Lower is better	16.25	22.34	20.32
Motricity Index	Right	Higher is better	44	48	56
Motricity Index	Left	Higher is better	52	56	56
Ashworth Scale	Right	Lower is better	6	6	5
Ashworth Scale	Left	Lower is better	6	6	5

Overall, the clinical findings suggest that our rehabilitation program was associated with improvements in balance, strength, and tone, whereas gait-related performance outcomes showed delayed rather than immediate benefit.

### 4.2 Instrumental gait and posturographic analysis

Instrumental gait analysis revealed time-dependent and side-specific changes. Step length decreased at T1 on both sides and increased at T2, with values at T2 approaching the normative reference range reported for the healthy cohort (12, 13). Cadence exhibited a slight net increase by T2 but remained markedly below normative values.

Global gait quality indices showed persistent asymmetry. On the right side, Gait Profile Score decreased and Gait Deviation Index increased at T2 compared with T0 and/or T1, whereas on the left side Gait Profile Score remained elevated and Gait Deviation Index remained below reference values at T2 (see Table 2). Step width also followed a non-linear pattern, increasing markedly at T1 and then improving at T2 (see Table 3).

**Table 3:** Instrumental outcomes variations across baseline (T0), post-Alter G only training (T1) and post-combined training (T2).

Outcome	T0	T1	T2	Normative
Gait analysis outcomes				
Step width (m)	0.12 ± .04	0.36 ± .24	0.1 ± .02	0.08 ± 0.05
Cadence (steps/min)	47.1 ± 7.729	39.9 ± 5.594	48.2 ± 3.493	114 ± 4.2
Gait Profile Score R	17 ± 2	19.3 ± 3.5	14.3 ± .4	<7
Gait Profile Score L	16.3 ± .9	20 ± 1.9	19 ± .4	<7
Gait deviation index R	68.08 ± 3.88	65.24 ± 5.66	75.29 ± .87	>100
Gait deviation index L	72.42 ± 2.26	64.39 ± 2.9	67.77 ± 1.91	>100
Step length (m) R	0.53 ± .05	0.26 ± .27	0.59 ± .05	0.62 ± 0.05
Step length (m) L	0.42 ± .16	0.27 ± .028	0.59 ± .04	0.62 ± 0.05
Posturography outcomes				
% load distribution—R foot—EO	46	48	50	
% load distribution—R foot—EC	43	40	41	
% load distribution—L foot—EO	54	52	50	
% load distribution—L foot—EC	57	60	59	
95% confidence ellipse—EO	405	1,750	1,147	
95% confidence ellipse—EC	754	958	468	
Center of Pressure (CoP) length—EO	101	241	147	
Center of Pressure (CoP) length—EC	133	108	129	
Average velocity COP—EO	10	24	15	
Average velocity COP—EC	13	11	13	

R, right; L, left; EO, eyes open; EC, eyes closed; CoP, center of pressure.

Taken together, gait parameters showed a non-linear pattern across timepoints, with marked changes at T1 and a partial shift toward baseline/reference values at T2, more evident on the right side.

Static posturography showed progressive normalization of foot load symmetry under eyes-open conditions, reaching a near 50/50 distribution at T2. Under eyes-closed conditions, however, asymmetry persisted, suggesting continued reliance on visual input for postural control. Confidence ellipse, center-of-pressure length, and average center-of-pressure velocity showed mixed trajectories. Some parameters worsened at T1 and improved at T2, whereas others remained higher than baseline, particularly under eyes-open conditions. Under eyes-closed conditions, selected parameters improved by T2, suggesting partial adaptation in sensory-challenged balance control (see Table 2). Overall, posturographic data support improved static load symmetry under visual guidance, but incomplete normalization of postural control.

### 4.3 Session-level training parameters

Session-level monitoring showed overall good physiological tolerance to treadmill training across both treatment blocks. Peripheral oxygen saturation remained stable and consistently within the normal range throughout the intervention (see Table 3). Heart rate and blood pressure showed only modest pre–post fluctuations, without clinically relevant instability (see Supplementary Figure S2). Compared with the Alter-G-only block, the combined block was associated with higher perceived exertion (4.70 ± 0.48 vs. 3.10 ± 1.79), lower mean distance per session (350.00 ± 104.77 m vs. 551.00 ± 175.34 m), and lower estimated energy expenditure (57.70 ± 10.45 kcal vs. 78.10 ± 9.85 kcal). In parallel, the combined phase was characterized by lower mean unweighting and greater incline, suggesting a training condition that was biomechanically more demanding despite lower walking volume.

Taken together, these session-level data suggest that the intervention was safe and physiologically manageable, while also indicating that the combined phase may have imposed a higher perceived effort despite lower average distance and caloric expenditure (See Supplementary Table S4).

## 5 Discussion

This case report describes the multidomain evolution of a patient with PLP1-related spastic paraparesis who underwent a sequential technology-assisted rehabilitation program composed of anti-gravity treadmill training followed by combined anti-gravity treadmill and wearable electrical stimulation. Clinically, we observed improvements in balance, lower limb strength, and spasticity by the end of treatment, along with non-linear changes in mobility, walking performance, and endurance, with initial worsening after the first block followed by partial recovery. Regarding instrumental parameters, we found positive results in step length and partial improvement in selected gait quality indices, particularly on the right side as well as improved load symmetry during quiet stance under eyes-open conditions, with persistent instability under sensory challenge.

To the best of our knowledge, this case represents one of the few available reports describing a structured rehabilitation technology-based intervention in an adult patient with PLP1-related spastic paraparesis. This is particularly relevant because rehabilitation evidence in PLP1-related disorders remains scarce, and most available data focus on diagnostic, genetic, pediatric, or supportive-care aspects rather than on quantified adult rehabilitation outcomes (1, 3, 20, 21). Overall, available therapeutic and rehabilitative approaches remain largely supportive and include conventional physiotherapy (1, 3, 4, 22), occupational therapy (22), orthotic management (4), pharmacological and focal spasticity treatment (23), botulinum toxin injections (24, 25), orthopedic or neurosurgical procedures (22, 26), treadmill- or robot-assisted gait rehabilitation (27, 28, 31), and wearable electrical stimulation systems (4). However, structured adult PLP1-specific rehabilitation protocols with multidomain clinical and instrumental outcomes remain largely lacking. In this context, the present case expands the existing literature by describing a sequential technology-assisted intervention combining anti-gravity treadmill training and wearable electrical stimulation, together with clinical, gait, posturographic, and session-level monitoring. A narrative summary of previously reported or proposed therapeutic and rehabilitative strategies for PLP1-related disorders and related hereditary spastic or hypomyelinating conditions is provided in Supplementary Table S5.

From a rehabilitation perspective, the observed pattern is clinically plausible. Body weight-supported treadmill training may have enabled intensive, task-specific gait practice while reducing mechanical demand and fall risk, which is particularly relevant in patients with spastic gait, poor foot clearance, and recurrent falls (7, 29). The progressive increase in treadmill demand through reduced unweighting and modest increases speed and incline may have promoted locomotor adaptation while maintaining safety.

During the second treatment block, the program was continued with AlterG while sessions with the EXOPULSE Mollii Suit were introduced. Although several changes (e.g., spasticity and fatigue-related measures) were temporally observed during this phase, the present sequential, uncontrolled design does not allow attribution of these changes to the electrosuit specifically. While a complementary role of wearable stimulation on tolerability or motor control has been hypothesized in the literature (9, 30), such an effect cannot be demonstrated in the present case. These findings should therefore be interpreted as an association with the overall intervention course, and alternative explanations, such as cumulative training dose, delayed adaptation to treadmill training, natural variability, or nonspecific contextual effects, remain equally plausible. Future studies using controlled designs (e.g., single-case experimental designs or randomized protocols) are needed to clarify whether wearable stimulation provides incremental benefit beyond treadmill-based training.

A relevant feature of this case is the discrepancy between clinical gains in balance and strength and the more complex trajectory of gait-related performance measures. We propose the hypothesis that, in chronic spastic gait disorders, reduced reliance on compensatory strategies may transiently worsen functional performance before more efficient motor patterns emerge. In this patient, the history of prior botulinum toxin treatment and the reported increase in fatigability after injection may be consistent with a close interplay between compensation and impairment. Accordingly, this hypothesis could account for the observed non-linear pattern, with some outcomes worsening after the first block before improving later; however, this interpretation remains hypothesis-generating and cannot be verified within a single-case design.

Instrumental gait analysis added important nuances to the interpretation of clinical change. Although cadence remained markedly abnormal, step length increased at T2 and approached normative values on both sides (Table 3), consistent with improvement in selected spatiotemporal features (21). Nevertheless, gait quality remained clearly pathological overall, especially on the left side, indicating that the intervention did not normalize gait but may have improved selected aspects of locomotor organization. Similarly, posturography suggested better static symmetry under visual conditions, whereas residual asymmetry under eyes-closed conditions pointed to persistent deficits in sensory integration or internal postural control. However, mechanistic inferences regarding sensory integration should be considered speculative.

An additional strength of this case is the availability of session-level physiological and training data collected during Alter-G sessions. Stable oxygen saturation and the absence of major cardiovascular fluctuations support the safety and tolerability of the intervention in this patient. Interestingly, the combined Alter-G plus wearable stimulation block was associated with higher perceived exertion despite lower average distance covered and lower estimated energy expenditure per session. One possible interpretation is that the addition of wearable stimulation increased subjective task demand, even when external walking volume was lower. In line with the hypothesis above, this pattern could reflect a transition from highly compensatory ambulation toward a more controlled but initially more effortful motor strategy. However, this remains speculative and should be interpreted cautiously.

Wearable multi-site low-frequency electrical stimulation such as the EXOPULSE Mollii Suit has been hypothesized to influence motor output through peripheral afferent input and, potentially, broader neuromodulatory pathways. A recent case report in multiple sclerosis (32) reported concurrent changes in clinical tone measures, neurophysiological and autonomic markers, suggesting that both peripheral and central processes may contribute. However, in the present case, no neurophysiological recordings were collected, and therefore any reference to cortical network modulation or supraspinal mechanisms must be considered theoretical. The observed reductions in spasticity, improvements in fatigue-related measures, and the non-linear trajectory of gait parameters after the combined block may be consistent with a contribution of peripheral afferent input to motor control, but they do not allow mechanistic inferences. Accordingly, this interpretation should be regarded as hypothesis-generating rather than evidence of a demonstrated mechanism in PLP1-related spastic paraparesis.

The present report contributes to the limited rehabilitation literature on PLP1-related disorders by highlighting the feasibility of a structured, technology-assisted intervention in an adult patient with preserved but vulnerable ambulatory function. The case may be particularly relevant because most available reports concern pediatric presentation, diagnostic issues, or supportive management rather than quantified rehabilitation outcomes.

This report has several limitations that should be considered when interpreting the findings. First, it describes a single patient and therefore has limited generalizability. Second, the study lacks a control condition and follows a sequential, uncontrolled design without washout, reversal, or randomization; therefore, causal inference is not possible, and the relative contribution of each intervention component (AlterG progression vs. wearable stimulation) cannot be unraveled.

Third, fatigue-related measures were collected only during the second treatment block, precluding a full baseline-to-post longitudinal interpretation. Fifth, the patient had undergone previous treatments (i.e., regular physiotherapy, botulinum toxin injections, and prior surgery) which may have influenced motor performance and adaptation over time and should therefore be considered potential confounders. Finally, the absence of post-intervention follow-up assessments prevents conclusions regarding the durability of the observed changes. Overall, these limitations indicate that the present findings should be considered exploratory and hypothesis-generating, primarily supporting feasibility and multidomain outcome monitoring in a rare neurological condition. These preliminary observations may inform future prospective studies in rare hypomyelinating and hereditary spastic disorders.

## 6 Conclusion

In this patient with PLP1-related spastic paraparesis, a sequential rehabilitation program based on anti-gravity treadmill training followed by combined anti-gravity treadmill plus wearable electrical stimulation was feasible and well-tolerated. Across timepoints, the intervention was accompanied by heterogeneous changes in clinical and instrumental outcomes that were not uniformly positive, with transient worsening in some gait- and endurance-related measures and partial recovery by the end of the program.

Given the single-case, sequential and uncontrolled design, these findings do not allow causal inference or any conclusion regarding the comparative effectiveness or superiority of one treatment block over the other. Rather, they should be considered exploratory and hypothesis-generating, supporting further investigation of multimodal, technology-assisted rehabilitation strategies in rare hereditary spastic disorders using controlled or single-case experimental designs.

## Data Availability

The datasets presented in this article are not readily available because of ethical and privacy restrictions. Requests to access the datasets should be directed to the corresponding author/s.
