# Supplementary material for: Multimodal rehabilitation in PLP1-associated spastic paraparesis: a case report with clinical and biomechanical outcomes
Source: Front Rehabil Sci. 2026 Jun 16;7:1837911. doi: 10.3389/fresc.2026.1837911 (PMC13314760; doi:10.3389/fresc.2026.1837911)
Supplement: Supplementary file 1 [file Table1.docx]

**Supplementary Table S1. EXOPULSE Mollii Suit stimulation settings.**

Settings are reported as the device ordinal value and the corresponding pulse width (µs). Conversion rule: value 1 = 25 µs, and each incremental unit corresponds to +5 µs (max value 30 = 170 µs).

| **Muscle/Region** | **Side** | **Setting value** | **Pulse width (µs)** |
| --- | --- | --- | --- |
| Anterior shoulder / upper chest region | R | 2 | 30 |
| Anterior shoulder / upper chest region | L | 2 | 30 |
| Upper arm (lateral/anterior region) | R | 4 | 40 |
| Upper arm (lateral/anterior region) | L | 4 | 40 |
| Forearm (anterior region) | R | 1 | 25 |
| Forearm (anterior region) | L | 1 | 25 |
| Abdominal wall (rectus region) | R | 6 | 50 |
| Abdominal wall (rectus region) | L | 6 | 50 |
| Central lower abdomen | Midline | 1 | 25 |
| Lateral trunk / abdominal oblique region | R | 9 | 65 |
| Lateral trunk / abdominal oblique region | L | 9 | 65 |
| Anterior thigh (diagonal electrode region) | R | 1 | 25 |
| Anterior thigh (diagonal electrode region) | L | 1 | 25 |
| Tibialis anterior | R | 21 | 125 |
| Tibialis anterior | L | 18 | 110 |
| Upper trapezius / neck region | R | 3 | 35 |
| Upper trapezius / neck region | L | 3 | 35 |
| Paraspinal / thoracic extensor region | R | 6 | 50 |
| Paraspinal / thoracic extensor region | L | 6 | 50 |
| Upper arm (posterior region) | R | 1 | 25 |
| Upper arm (posterior region) | L | 1 | 25 |
| Forearm (posterior region) | R | 9 | 65 |
| Forearm (posterior region) | L | 12 | 80 |
| Gluteal region (diagonal electrodes) | R | 1 | 25 |
| Gluteal region (diagonal electrodes) | L | 1 | 25 |
| Lateral hip region | R | 15 | 95 |
| Lateral hip region | L | 15 | 95 |
| Posterior thigh region | R | 9 | 65 |
| Posterior thigh region | L | 9 | 65 |
| Posterior calf region | R | 1 | 25 |
| Posterior calf region | L | 1 | 25 |
