# Supplementary material for: Multimodal rehabilitation in PLP1-associated spastic paraparesis: a case report with clinical and biomechanical outcomes
Source: Front Rehabil Sci. 2026 Jun 16;7:1837911. doi: 10.3389/fresc.2026.1837911 (PMC13314760; doi:10.3389/fresc.2026.1837911)
Supplement: Supplementary file 2 [file Table2.docx]

**Supplementary table 2**

**Supplementary Table 2. Description of the clinical tools used to evaluate the patient.**

| Test/Scale | Domain | Description |
| --- | --- | --- |
| FES-I | Fear of falling | The FES-I (Falls Efficacy Scale – International) is a scale that assesses fear of falling during 16 daily activities, with scores ranging from 1 (not at all concerned) to 4 (very concerned). The total score, from 16 to 64, reflects the level of fall-related anxiety and is useful for identifying at-risk individuals and monitoring fall-prevention interventions. |
| BBS | Balance | The BBS (Berg Balance Scale) is a clinical tool used to assess static and dynamic balance in adults, especially the elderly or neurological patients. It includes 14 daily activities, such as standing, turning, reaching, and stair climbing. Each task is scored from 0 to 4, with a maximum total of 56, where lower scores indicate a higher risk of falling. |
| TUG | Mobility | The TUG (Timed Up and Go) test assesses mobility, balance, and fall risk. The patient stands up from a chair, walks 3 meters, turns, walks back, and sits down. The time taken to complete the task is recorded, with longer times indicating reduced mobility and higher fall risk. The test was performed twice, once including a right turn and once including a left turn. These are reported as TUG R and TUG L, respectively, to quantify potential asymmetries in turning performance |
| MI | Motor strength | The Motricity Index (MI) is a clinical scale used to assess limb motor strength in patients with neurological impairments. It evaluates key movements of the upper and lower limbs, scoring each from 0 (no movement) to 33 (normal strength), with a total score reflecting overall motor function. |
| ASHWORTH  SCALE | Spasticity | The AS (Ashworth Scale) assesses muscle spasticity by rating resistance during passive stretching of a muscle. Scores range from 0 (no increase in tone) to 4 (limb rigid in flexion or extension). For each lower limb, MAS was rated at three joints (hip, knee, and ankle/foot), and a composite MAS score was calculated as the sum of the three joint scores. |
| 10 MWT | Gait speed | The 10MWT assesses short-distance walking performance over 10 meters and is commonly used to quantify gait speed and functional mobility. In the present case report, results are reported as completion time (seconds), with lower values indicating better performance. Although the 10MWT is often expressed as walking speed (m/s), we presented the raw time to maintain consistency with other time-based clinical measures (e.g., the TUG). |
| 6 MWT | Endurance | The 6MWT (6 Minute Walk Test) evaluates endurance and functional exercise capacity by measuring the distance a person can walk in 6 minutes. Longer distances reflect better cardiovascular and muscular function. |
| VAFS | Fatigue | The VAFS (Visual Analog Fatigue Scale) assesses subjective fatigue by having patients mark their fatigue level on a line, typically 0 (no fatigue) to 10 (maximum fatigue). |
| FSS | Fatigue | The FSS is a 9-item self-administered questionnaire designed to evaluate the severity of fatigue and its interference with physical, social, and functional activities. Each item is scored from 1 to 7, with higher scores reflecting greater fatigue severity. |

**Legend:** FES-I, Falls Efficacy Scale–International; BBS, Berg Balance Scale; TUG, Timed Up and Go; Motricity Index, Motricity Index;10MWT, 10-Meter Walk Test; 6MWT, 6-Minute Walk Test; VAFS, Visual Analog Fatigue Scale; FSS, Fatigue Severity Scale.
