# Supplementary material for: Multimodal rehabilitation in PLP1-associated spastic paraparesis: a case report with clinical and biomechanical outcomes
Source: Front Rehabil Sci. 2026 Jun 16;7:1837911. doi: 10.3389/fresc.2026.1837911 (PMC13314760; doi:10.3389/fresc.2026.1837911)
Supplement: Supplementary file 3 [file Table3.docx]

**Table S3.** Published measurement error estimates (MDC95/SEM) used for exploratory contextualization of change. The table reports the minimal detectable change at the 95% confidence level (MDC95) (or SEM-derived MDC95 when only SEM was available) for selected clinical outcomes, together with the source population and reference for each value. Because disease-specific measurement properties for PLP1-related spastic paraparesis are not available and most estimates derive from stroke or other clinical populations, these values were used solely as a rough, descriptive reference to contextualize observed changes and were not used to draw inferential conclusions.

| Outcome | Side | Direction of improvement | MDC95-based contextualization of change | | | References |
| --- | --- | --- | --- | --- | --- | --- |
|  |  |  | **T0-T1** | **T1-T2** | **T0-T2** |  |
| FES-I | — | Lower is better | 0.000 | 0.000 | 0.000 | 15 |
| Berg Balance Scale (BBS) | — | Higher is better | 0.475 | 1.424 | 1.898 | 17 |
| 10-Meter Walk Test (s) | — | Lower is better | -5.650 | 3.793 | -1.858 | 16 |
| 6-Minute Walk Test (m) | — | Higher is better | -1.263 | 1.010 | -0.253 | 14 |
| Fatigue Severity Scale (FSS) | — | Lower is better | — | -2.450 | — | 19 |
| Visual Analog Fatigue Scale (VAFS) | — | Lower is better | — | NA | — | 19 |
| Timed Up and Go (TUG, s) | Right | Lower is better | 2.420 | -2.447 | -0.027 | 16 |
| Timed Up and Go (TUG, s) | Left | Lower is better | 4.116 | -1.366 | 2.751 | 16 |
| Motricity Index | Right | Higher is better | 0.607 | 1.213 | 1.820 | 18 |
| Motricity Index | Left | Higher is better | 0.607 | 0.000 | 0.607 | 18 |
| Ashworth Scale | Right | Lower is better | NA | NA | NA |  |
| Ashworth Scale | Left | Lower is better | NA | NA | NA |  |
