# Supplementary material for: Multimodal rehabilitation in PLP1-associated spastic paraparesis: a case report with clinical and biomechanical outcomes
Source: Front Rehabil Sci. 2026 Jun 16;7:1837911. doi: 10.3389/fresc.2026.1837911 (PMC13314760; doi:10.3389/fresc.2026.1837911)
Supplement: Supplementary file 4 [file Table4.docx]

**Table S4.** Session-level training parameters across each training block and for the overall training period. Values are reported as mean ± SD across sessions within each block (sessions 1–10: AlterG-only; sessions 11–20: combined), and across the full training period (overall: all 20 sessions).

| Parameter | Alter-G only (1–10), mean ± SD | Combined (11–20), mean ± SD | Overall (1–20), mean ± SD |
| --- | --- | --- | --- |
| Pre systolic BP (mmHg) | 114.90 ± 6.19 | 115.60 ± 9.03 | 115.25 ± 7.55 |
| Post systolic BP (mmHg) | 125.60 ± 4.95 | 118.10 ± 5.95 | 121.85 ± 6.57 |
| Pre diastolic BP (mmHg) | 60.70 ± 5.21 | 69.80 ± 7.94 | 65.25 ± 8.03 |
| Post diastolic BP (mmHg) | 68.70 ± 3.80 | 66.20 ± 5.20 | 67.45 ± 4.62 |
| Pre heart rate (bpm) | 76.40 ± 8.87 | 75.30 ± 11.65 | 75.85 ± 10.10 |
| Post heart rate (bpm) | 79.50 ± 4.79 | 81.30 ± 8.90 | 80.40 ± 7.01 |
| Pre SpO₂ (%) | 98.40 ± 0.52 | 98.30 ± 0.67 | 98.35 ± 0.59 |
| Post SpO₂ (%) | 98.40 ± 0.52 | 98.60 ± 0.52 | 98.50 ± 0.51 |
| Speed (km/h) | 0.66 ± 0.23 | 0.56 ± 0.11 | 0.61 ± 0.18 |
| RPE | 3.10 ± 1.79 | 4.70 ± 0.48 | 3.90 ± 1.52 |
| Energy expenditure (kcal) | 78.10 ± 9.85 | 57.70 ± 10.45 | 67.90 ± 14.39 |
| Distance per session (m) | 551.00 ± 175.34 | 350.00 ± 104.77 | 450.50 ± 174.34 |
