# Supplementary material for: Multimodal rehabilitation in PLP1-associated spastic paraparesis: a case report with clinical and biomechanical outcomes
Source: Front Rehabil Sci. 2026 Jun 16;7:1837911. doi: 10.3389/fresc.2026.1837911 (PMC13314760; doi:10.3389/fresc.2026.1837911)
Supplement: Supplementary file 5 [file Table5.docx]

**Supplementary Table S5.** Therapeutic and rehabilitative strategies reported or proposed for PLP1-related disorders and related hereditary spastic/hypomyelinating conditions

| Therapeutic / rehabilitative strategy | Clinical rationale / target | Evidence and reported use in the literature | Main limitations or unresolved issues | Relevance to the present case |  |
| --- | --- | --- | --- | --- | --- |
| Conventional physiotherapy, occupational therapy, stretching, strengthening, balance training, and functional exercise | Maintenance of mobility, prevention of contractures, improvement of strength, balance, transfers, and activities of daily living (3,4,21,22). | PLP1-related disorders are generally managed through supportive multidisciplinary care, including physical therapy, exercise, occupational therapy, adaptive devices, and orthotic management. A pediatric PMD case report (3) described developmental improvement after structured rehabilitation, but PLP1-specific adult rehabilitation evidence remains scarce. | Most reports are pediatric or supportive-care oriented. Protocol intensity, progression criteria, and standardized multidomain outcomes are rarely described, especially in adults with PLP1-related spastic paraparesis. | The patient had previously received conventional physiotherapy, but gait impairment, instability, and recurrent falls persisted. The present intervention expands beyond conventional therapy by adding structured technology-assisted gait training and objective instrumental monitoring. | |
| Orthoses, assistive devices, and adaptive equipment | Compensation for weakness, equinus, instability, or impaired foot clearance; prevention of falls and secondary musculoskeletal complications. | Orthotics and adaptive devices are recommended as part of routine supportive management in PLP1-related disorders, particularly for spasticity, ataxia, joint contractures, scoliosis, and functional mobility limitations (4). | These approaches may improve safety and compensation but do not directly target locomotor relearning or neuromuscular adaptation. Evidence is usually descriptive rather than based on controlled rehabilitation protocols. | The patient occasionally used a forearm crutch for safety but preferred not to rely on it. The present approach aimed to promote active gait practice rather than only compensate for gait impairment. |  |
| Pharmacological spasticity management | Reduction of generalized spasticity and muscle overactivity; improvement of comfort, hygiene, posture, and mobility. | Symptomatic spasticity management in PLP1-related disorders may include medications such as baclofen, diazepam, and tizanidine. Similar symptomatic approaches are commonly described in hereditary spastic paraplegia (23). | Pharmacological treatment may be limited by sedation, weakness, variable functional response, and lack of disease-modifying effect. Improvements in tone do not necessarily translate into better gait. | The present case did not test pharmacological treatment. Instead, it explored whether task-specific technology-assisted rehabilitation could improve selected motor domains while preserving active locomotor participation. |  |
| Botulinum toxin treatment combined with physiotherapy | Focal reduction of overactive muscles, particularly adductors, hamstrings, rectus femoris, gastrocnemius, or soleus, to improve gait mechanics and reduce spasticity. | In HSP, BoNT-A combined with intensive physiotherapy has been associated with improvement in muscle tone, gait velocity, walking distance, pain, and quality of life. However, systematic review evidence also suggests that spasticity reduction may sometimes uncover weakness or modify compensatory gait strategies (24-25). | Evidence is mostly from HSP rather than PLP1-specific cohorts. Functional response is variable and depends on muscle selection, dosage, baseline weakness, compensatory strategies, and post-injection rehabilitation. | The patient had previously received incobotulinumtoxinA injections to bilateral gastrocnemius medialis/lateralis and soleus muscles, followed by increased fatigability and no clear qualitative gait improvement. This supports the need for cautious interpretation of tone reduction in chronic compensatory spastic gait. |  |
| Orthopedic or neurosurgical interventions | Management of fixed contractures, deformities, severe scoliosis, focal spastic patterns, or refractory musculoskeletal complications. | Surgery for joint contractures or severe scoliosis is included among supportive management options in PLP1-related disorders. Non-pharmacological HSP literature also includes surgical approaches among possible interventions (22,26). | Surgical or neuro-orthopedic approaches may address focal structural or tone-related problems but do not directly restore gait control. Effects may be irreversible and must be integrated with rehabilitation. | The patient had previously undergone neurotomy of the motor branches of the obturator nerve targeting hip adductors. |  |
| Treadmill-based, body-weight-supported, or robot-assisted gait rehabilitation | Intensive, repetitive, task-specific gait practice with reduced fall risk and controlled mechanical loading. | In HSP, gait rehabilitation and robotic gait training have been explored in small studies and case reports. One adult HSP case report using robot-assisted gait training plus physiotherapy reported improved walking speed and balance, although gait kinematics and kinetics did not markedly change. Systematic reviews highlight that intervention evidence remains limited and heterogeneous (29-30). | Evidence is largely non-PLP1-specific. The optimal dose, progression criteria, and long-term effects remain unclear. Robotic or treadmill-based interventions may improve selected functional outcomes without normalizing gait quality. | The present case used an anti-gravity treadmill rather than robotic assistance. AlterG training allowed progressive modulation of body-weight support, speed, and incline while maintaining safety in a patient with falls and severe gait impairment. |  |
| Wearable electrical stimulation systems / multisite neuromodulation, including EXOPULSE Mollii Suit | Modulation of spasticity, facilitation of voluntary movement, afferent sensory input, and possible improvement in fatigue, pain, or motor control. | The EXOPULSE Mollii Suit is a full-body transcutaneous electrical stimulation system. In chronic stroke, a single 60-minute session did not consistently reduce objective spasticity at group level, although individual responses were variable. More recent MS evidence suggests potential benefits on balance, spasticity, mobility, fatigue, and quality of life, but larger and longer-term studies are needed (24, 34). | Evidence is condition-specific and heterogeneous. Mechanisms remain uncertain, and additive effects beyond physiotherapy or treadmill training are not yet established. Wearing the suit may also require assistance in some patients. | In the present case, wearable stimulation was added only during the second block. Therefore, changes observed after this phase cannot be attributed specifically to the suit, but the case provides preliminary feasibility data for combining wearable stimulation with anti-gravity treadmill training in PLP1-related spastic paraparesis. |  |
