# Supplementary material for: Multimodal rehabilitation in PLP1-associated spastic paraparesis: a case report with clinical and biomechanical outcomes
Source: Front Rehabil Sci. 2026 Jun 16;7:1837911. doi: 10.3389/fresc.2026.1837911 (PMC13314760; doi:10.3389/fresc.2026.1837911)
Supplement: Supplementary file 6 [file Datasheet1.docx]

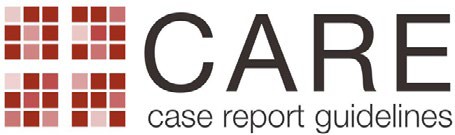

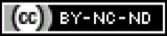

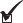
**CARE Checklist of information to include when writing a case report**

| **Topic** | **Item No** | **Checklist item description** | **Reported on Section/Paragraph** |
| --- | --- | --- | --- |
| Title | 1 | The diagnosis or intervention of primary focus followed by the words “case report” | Title |
| Key Words | 2 | 2 to 5 key words that identify diagnoses or interventions in this case report, including "case report" | Keywords |
| Abstract  (Structured summary) | 3a | Background: state what is known and unknown; why the case report is unique and what it adds to existing literature. | Abstract |
|  | 3b | Case Description: describe the patient’s demographic details, main symptoms, history, important clinical findings, the main diagnosis, interventions, outcomes and follow-ups. | Abstract |
|  | 3c | Conclusions: summarize the main take-away lesson, clinical impact and potential implications. | Abstract |
| Introduction | 4 | One or two paragraphs summarizing why this case is unique **(may include references)** | Introduction |
| Patient Information | 5a | De-identified patient specific information | Case Presentation |
|  | 5b | Primary concerns and symptoms of the patient | Case Presentation |
|  | 5c | Medical, family, and psycho-social history including relevant genetic information | Case Presentation |
|  | 5d | Relevant past interventions with outcomes | Case Presentation; Previous therapeutic management |
| Clinical Findings | 6 | Describe significant physical examination (PE) and important clinical findings | Functional Diagnosis and Rehabilitation Rationale; Clinical presentation at admission |
| Timeline | 7 | Historical and current information from this episode of care organized as a timeline | Case presentation + Table 1 |
| Diagnostic Assessment | 8a | Diagnostic testing (such as PE, laboratory testing, imaging, surveys). | Diagnostic assessment + Table 1 |
|  | 8b | Diagnostic challenges (such as access to testing, financial, or cultural) | Diagnostic assessment + Table 1 |
|  | 8c | Diagnosis (including other diagnoses considered) | Diagnostic assessment + Table 1 |
|  | 8d | Prognosis (such as staging in oncology) where applicable | NA |
| Therapeutic Intervention | 9a | Types of therapeutic intervention (such as pharmacologic, surgical, preventive, self-care) | Materials and Methods; Intervention |
|  | 9b | Administration of therapeutic intervention (such as dosage, strength, duration) | Materials and Methods; Intervention |
|  | 9c | Changes in therapeutic intervention (with rationale) | Intervention; Results;  Discussion |

| Follow-up and Outcomes | 10a | Clinician and patient-assessed outcomes (if available) |  |  |
| --- | --- | --- | --- | --- |
|  | 10b | Important follow-up diagnostic and other test results |  |  |
|  | 10c | Intervention adherence and tolerability (How was this assessed?) |  |  |
|  | 10d | Adverse and unanticipated events |  |  |
| Discussion | 11a | A scientific discussion of the strengths AND limitations associated with this case report |  |  |
|  | 11b | Discussion of the relevant medical literature **with references** |  |  |
|  | 11c | The scientific rationale for any conclusions (including assessment of possible causes) |  |  |
|  | 11d | The primary “take-away” lessons of this case report (without references) in a one paragraph conclusion |  |  |
| Patient Perspective | 12 | The patient should share their perspective in one to two paragraphs on the treatment(s) they received |  |  |
| Informed Consent | 13 | Did the patient give informed consent? Please provide if requested | **Yes** | **No** |

Please leave this space alone as it will be supplemented by the editorial office when needed.
